# Defining Pooled’ Place-Based’ Budgets for Health and Social Care: A Scoping Review

**DOI:** 10.5334/ijic.6507

**Published:** 2022-09-13

**Authors:** Davide Tebaldi, Jonathan Stokes

**Affiliations:** 1 Health Organisation, Policy & Economics (HOPE), Centre for Primary Care and Health Services Research, University of Manchester, Oxford Road, Manchester M13 9PL, England

**Keywords:** pooled budgets, place-based budgets, integrated care, scoping review

## Abstract

**Introduction:**

Current descriptions of pooled budgets in the literature pose challenges to good quality evaluation of their contribution to integrated care. Addressing this gap is increasingly important given the shift from early models of integrated care targeting segments of the population, to more recent approaches that aim to target ‘places’, broader geographically defined populations. This review draws on the current international evidence to describe practical examples of pooled health and social care budgets, highlighting specific place-based approaches.

**Methods:**

We initially conducted a scoping review, a systematic database search (‘Medline’, ‘Embase’, ‘Econ Lit’ and ‘Google Scholar’) complemented by further snowballing for academic and ‘grey literature’ publications (1995 – 2020). Results were analysed thematically according to budget characteristics and macro-environment, with additional specific case studies.

**Results:**

Thirty-six primary studies were included, describing ten broad models of pooled budgets across seven countries. Most budgets targeted specific sub-populations rather than an entire geographically defined population. Specific budget structures varied and were generally under-described. The closest place-based models were for small populations and implemented in a national health system, or insurance-based with natural geographical boundaries.

**Conclusion:**

Despite their increasing relevance in the current political debate, pooled place-based budgets are still at an early stage of implementation and research. Adequate description is required for future meta-analysis of effectiveness on outcomes.

## Introduction

The lack of integration of budgets across the health and social care sectors is a potential barrier to better-integrated care [1,2,3]. When there are two or more providers of different services to an individual (‘user’), those services interact to define the overall benefit to the user and indirectly influence each provider’s contribution to the health and social care system [1,4]. For example, inappropriate social care capacity will affect the user’s well-being and increase the risk of hospital admission (and possibly the cost) [5]. Equally, hospital care that reduces impairment may reduce future social care costs. The productivity of each provider is closely interdependent, and not considering this joint production of patient outcomes might represent a unique missed opportunity for the health and care systems [5,6,7,8].

In theory, pooled budgets could incentivise each organisation to consider outside of their very own ‘slice’ of budget activity. This could help sectors achieve shared goals more efficiently, for instance, by avoiding the duplication of services [9]. Therefore, in theory, pooled budgets are more likely to consider and produce holistic outcomes for the populations they serve. However, while several theories on the potential benefits of pooling budgets on health outcomes and service use exist [2,9,10,11,12,13], the vast majority of which are based on the economic concept of the agency theory, less convincing empirical evidence supports this theoretical prediction [2,10,14,15]. The agency theory predicts [2] that given the interdependencies between the health and social care sectors described above, it is unlikely that rewarding each provider separately for each package of care they produce will result in the best achievable health outcomes for patients. This is because in a single agency relationship each individual provider will have incentives to economise and thus to achieve the lowest cost of delivery consistent with its own goals. An alternative approach could be to align different providers incentives by tying them together financially with a single capitated budget covering both health and social care for each individual covered. In such a scenario, in absence of asymmetric behaviour and assuming a common governance and regulatory framework between providers, this kind of gaming behaviour should cease to exist. However, several systematic reviews summarised the effectiveness of pooled budgets [1,2,3,4,16,17,18,19,20,21,22,23,24,25,26,27,28] and, overall, despite some positive results, the evidence they produced remains sparse and still surrounded by numerous uncertainties [2,3,29,30,31,32]. These uncertainties related to pooled budgets in the empirical evidence are partly due to several nebulous implementations of the concept, with a high degree of heterogeneity [2,3]. What is more, the available academic literature has not been able to describe this heterogeneity [2,10] entirely, a first step to unpack ‘what works, in which circumstances and for ‘whom’. For instance, there is some evidence of unintended consequences associated with the implementation of pooled-budget initiatives such as premature discharging and increased risk or readmission but, overall, there is also a current lack of emphasis on what the role of the macro environment might be and on potential unintended consequences [2]. Therefore, there is a need to clarify how real-world examples of pooled budgets have been practically implemented internationally.

Furthermore, addressing this descriptive gap seems increasingly important, given the shift from early integrated care models, which were highly focused on a small number of high-cost/-risk individuals [33,34], to the more recent approaches which aim to target ‘places’ [31,35], broader geographically defined populations and towards incentivising more preventative, proactive care [36]. This shift is happening globally [35]. In England, for example, this change is clear from the Vanguard ‘New Care Models’, aimed at maximising the value for patients by promoting better integration between health and social care organisations [37], in line with the NHS long term plan, a comprehensive programme which was designed to tackle the growing demand for health and social care services [31,38,39,40]. The aforementioned change is also clear from the most recent reform of Integrated Care Systems (ICSs) [40], inspired by US Accountable Care Organisations, which aims to promote partnership of organization to deliver better joined up health and social care services. A recent White Paper related to ICS reform, published by the Department of Health and Social Care, highlights the importance of a place-based care system to deliver this change. It also announced a sizeable legislative change to remove barriers for more efficient joint working between the NHS, local government, community health services, and voluntary organisations at local and system levels [41]. Arguably, the pooling of budgets has increasingly become a political choice to align incentives among different partners forming the health and social care system. This political choice explicates either through national policies [6] or regional/local initiatives e.g. MESO-level integration [42]. This review addresses these gaps by scoping the available literature for practical examples of pooled budgets between health and social care, establishing critical criteria upon which to describe and differentiate these examples, and, among current examples, identifying which of them could be considered a whole ‘place-based’, pooled budget approach.

## Methods

Several reasons contributed to adopting the scoping review methodological framework [43,44,45]. Firstly, the heterogeneity, complexity and frequent lack of clarity in the description and definition of ‘pooled place-based budgets’ required a flexible and exploratory approach that can be difficult to achieve with a more traditional narrow-focused systematic review [43,44,45,46,47]. Secondly, the available literature around the topic is sparse and with a lot of the budget description occurring in the grey literature, such as reports, policy statements, and other non-academic sources [9,35,36,48,49,50], which demanded a more malleable approach able to incorporate evidence from a wide variety of different sources [46,51]. Thirdly, this research aimed to map conceptual boundaries and provide working definitions for ‘pooled place-based budgets’ rather than answering a specific pre-defined research question, such as whether these interventions effectively achieved better-integrated care.

Following the latest guidelines for conducting scoping reviews, the present study was developed in six stages, described below: (i) Identifying the research question, (ii) Identifying relevant studies, (iii) study selection, (iv) charting the data, (v) collating, summarizing, and reporting the results, (vi) consultation [44,46,47].

### Identifying the research question

To answer the main research question of what constitutes pooled place-based budgets, we first had to define a set of sub-questions against which the examples of pooled budgets identified in the literature were extracted and initially described. These were:

- Which budgets are being pooled? e.g., single services vs multiple services
- How much of the total budget is being pooled?
- At which population level? e.g. national, regional or neighbourhood level
- Is the budget fully place-based? i.e. is it allocated based on a geographical determination
- Does the macro-environment e.g. a set of economic, demographics, natural and social factors/condition that exists in a specific geographical area, affect the primary structure or boundaries and implementation?
- What are the reported intended and unintended consequences?

### Identifying relevant studies

We searched the published and grey literature for examples of pooled health and social care budgets. We included qualitative and quantitative studies and adopted a three-stage search strategy. In the first stage, we systematically searched relevant databases, including Medline, Embase, EconLit, and Google Scholar, by combining synonyms of “pooled budget”, “place-based budget”, and similar words related to the broad concept of integrating funds for health and social care. It is worth noting that Google Scholar was specifically included in the systematic search strategy for its ability to effectively capture the majority of the relevant grey literature on a topic of interest by simply using a single search facility rather than a plethora of individual websites [52]. As previous research has found, Google Scholar was able to retrieve a substantial amount of the relevant grey literature on a specific topic of interest [52]. Therefore, we hoped that the inclusion of this database would also be able to identify the relevant international grey literature on pooled health and social care budgets. The initial search strategy was tailored to identify exclusively existing review studies since we were aware of at least ten systematic reviews that had already examined financial mechanisms to promote inter-sectorial action for health, including pooled budgets. We identified further review articles from the grey literature by searching among the relevant databases of independent charities in the field of health and social care, the King’s Fund, the Social Care Institute for Excellence (SCIE) and the Health Foundation, all British institutions but with an interest in providing international evidence and examples for integrated care and other health and care system reforms. Secondly, we conducted a hand-search among the list of references identified within relevant reviews for the primary studies of interest. Finally, we attempted to identify any examples not included in the previous reviews through a further ‘snowballing search’ [53]. According to this approach, we iteratively searched for additional materials we had identified as included (e.g. qualitative studies providing more detail when a primary study identified was quantitative with fewer details of the budget itself) and additional examples referenced within included primary studies. This last stage allowed the flexibility to include primaries studies that, for various reasons, might not have been included among the records previously identified [53]. All the extracted articles were stored on the reference managing software Endnote X9. As follow the complete list of inclusion and exclusion criteria:

**Inclusion criteria:**

- Academic publications and non-peer-reviewed literature describing practical examples of previously separate budgets forming a ‘new’ pooled budget, as defined by Mason et al., 2015, i.e. each partner contributes to a common fund for spending on agreed projects and services to achieve shared outcomes. This definition does not imply that one of the partners must have been formerly focused on healthcare alone and the other on social care alone.
- The ‘new’ pooled budget is used to finance and/or provide, at least, health and social care services. Services of any scope, a single service or a broad set.
- Only articles with sufficient details to determine whether the pooled budget initiatives were practically implemented in the ‘real-world’ setting. Consequently, the articles must contain evidence that the ‘new’ budget as described above was directly used to finance and or provide, at least, health and social care services.
- English Language.
- Only studies published after 1995, given that the interest in how pooled budgets can promote intersectorial action for health, can largely be traced after that period.
- Evaluation (quantitative, qualitative, or mixed methods studies) available in the literature.

**Exclusion criteria:**

- Schemes that exclusively integrated non-financial resources, e.g. staff, facilities, equipment or know-how.
- Schemes where the management and accountability for the pooled budgets remained entirely separate, e.g. aligned budgets, lead commissioning.
- Sources lack details to determine whether the pooled-funding initiative passed the planning stage.
- Guidelines for how to implement pooled budgets (however, relevant primary sources were sourced in this case, where available).
- Articles that did not have, as their primary focus, the description of pooled budgets arrangements.
- Articles with insufficient information to determine whether they meet the inclusion criteria.

### Study selection

The attention was on the component and definition of ‘pooled’ and ‘place-based’ budgets for health and social care, focusing on the uncertainties related to the main research question. We concentrated on programmes or interventions that passed the planning stage and for which an evaluation, from the academic or non-academic literature, was already available.

### Mapping the data

Based on the literature’s initial screening, we iteratively developed an extraction template to classify the key characteristics of the examples of ‘pooled budgets’ and facilitate the comparison across their different components. As a result, we extracted the following information from the selected references included in the final qualitative synthesis:

- Title of the publication.
- First author and year
- Study design
- Country
- Country health system type [54]
- Target population
- Description of the budget
- Whether the pooled budget is also place-based, i.e. geographical component
- Summary of the study findings

### Collating, summarising and reporting the results

We analysed and reported the information from the selected studies to map the existing literature on ‘pooled’ and ‘place-based’ budgets for health and social care. We first reported the results from the search and screening, described above. We then summarised the main characteristics of the examples of pooled budgets that satisfied our list of selection criteria in a table. Subsequently, the summary information was analysed thematically according to each sub-question, shown above, and reported separately in two different subsections of the main results. The first subsection defines how the international examples of pooled budgets were described across the key domains in the literature. The last subsection focuses more on specific case study examples most consistent with the concept of ‘place based’ budgets. These case studies were intended to add more description of the specific cases of the ‘place based’ budgets, although there were too few examples to overly-generalise in relation to the research questions for these specific cases.

### Consultation

Finally, an initial report containing a summary of the preliminary findings of the current review was presented on two different occasions to local policymakers within the Greater Manchester devolved health and social care region with policymakers giving general feedback on the suitability of the findings in terms of policy applicability (details in the appendix).

## Results

**Figure 1:**
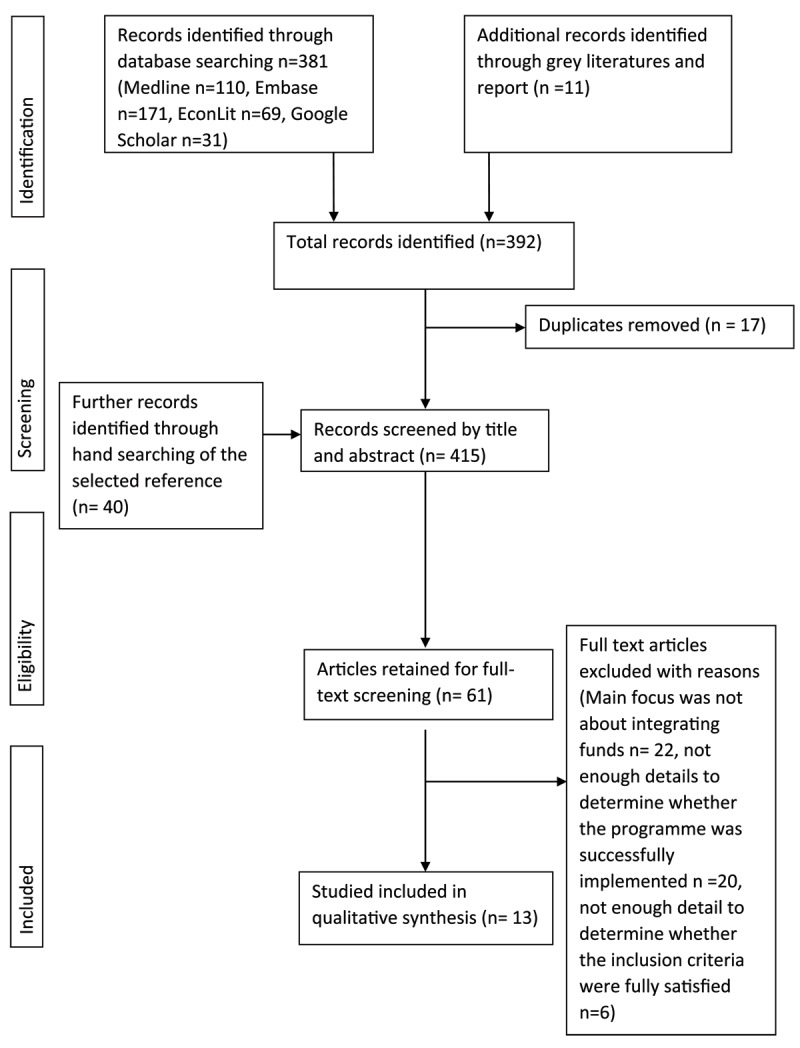
Flowchart diagram of the study selection process for the systematic search.

Figure 1 shows the flowchart diagram for the systematic search and screening of the included review studies. The initial database search identified 381 peer-reviewed academic studies. A further 11 grey-literature reports were retrieved. In total, 392 articles were identified at this stage. After 23 duplicates were removed and a further 40 records were retrieved by hand searching the selected references, one author DT proceeded to the title and abstract screening of the remaining 415 records. Therefore, the last count of 61 papers was eligible for full-text screening. Among these 61 records, 48 did not meet the inclusion criteria and were removed. Thirteen review studies satisfied all the inclusion criteria and were therefore included in the final qualitative synthesis [1,2,3,4,16,17,18,19,20,21,22,23,25,26,27,28].

**Figure 2:**
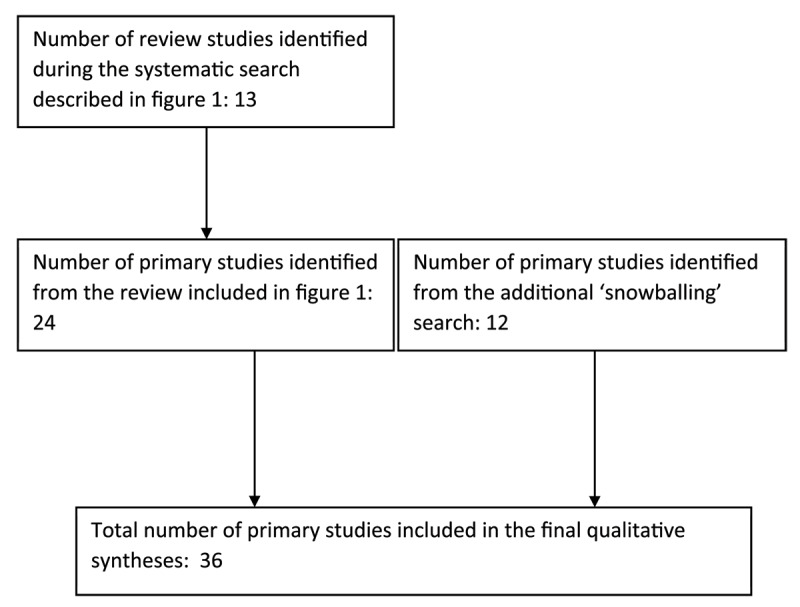
Flowchart diagram of the study selection process for the further ‘snowballing’ search.

We then identified and classified the relevant international examples of pooled budgets by looking directly at the sources of primary studies within the reviews. A total of 24 primary studies were identified at this stage [6,9,10,18,22,29,32,42,55,56,57,58,59,60,61,62,63,64,65,66,67,68,69,70,71,72,73,74,75,76,77,78,79,80,81,82,83,84,85,86,87,88]. Subsequently, the additional ‘snowballing’ search identified a further 12 primary studies [7,15,59,69,70,71,89,90,91,92,93,94,95,96,97,98]. Thirty-six primary studies were therefore included in the final synthesis (see Figure 2) [6,7,10,11,23,29,32,42,51,55,56,57,58,59,60,62,63,64,66,67,68,69,70,71,83,90,91,92,93,94,96,97,98,99,100,101,102,103,104,105,106,107,108,109,110,111,112,113,114,115,116,117,118].

Ten broad categories of pooled budget models across seven countries were identified (see Table 1). However, the description of the models was often generic and not reported in detail regarding their practical implementation elements. For example, it was not always straightforward to retrieve information about the size of the budget and if it was composed of additional or existing funds. Furthermore, very few examples could claim to be entirely place-based. Out of the ten broad examples of pooled budget initiatives, only three described a population-based approach.

**Table 1:** Summary of the models included in the final qualitative synthesis.


MODEL	COUNTRY	COUNTRY HEALTH SYSTEM TYPE ([54])	TARGET POPULATION	DESCRIPTION OF THE BUDGET	PLACE-BASED (YES/NO)	SUMMARY OF FINDINGS

1. Australian Coordinated Care Trials (ACT) [32,66,72,73,74,75].	Australia	**National Health Insurance** **Regulation:** State **Financing:** State **Provision:** Private	**Population size:** ~5000 participants for each of the nine trials. **Population description:** Patients with complex healthcare needs	**Monetary size:** Capitated budget of 0.50 to 5 Australian dollars per participant per day depending on which sites. **Existing/Additional funding:** Additional **Service included:** a broad set of services.	No	**Study Design:** Randomised controlled trial **Summary of findings:** cost-neutral and the mean quality of life did not improve.

2. SIPA (System of Integrated Services for Aged Persons) [9,18,22,68,73,83,84,85].	Canada	**National Health Insurance** **Regulation:** State **Financing:** State **Provision:** Private	**Population size:** 606 participants **Population description:** elderly persons.	**Monetary size:** capitated budget of $400 per patient per year. **Existing/Additional funding:** Additional **Service included:** a broad set of services	No	**Study Design:** Randomised controlled trial **Summary of findings** Accessibility increased, no significant difference in hospital services utilisation or costs.

3. The Program of All-inclusive Care of the Elderly (PACE) [76,77,78,79,80,81,119,120]	United States	**Private Health System** **Regulation:** Private **Financing:** Private **Provision:** Private	**Population size:** 651 participants. **Population description:** frail elderly persons	**Monetary size:** capitated budget. **Service included:** a broad set of services	No	**Study Design:** A cross-sectional time-series **Summary of findings:** Decreased hospital admission rates and hospital length of stay.

4. The British Columbia Model [61,86,87,121]	Canada	**National Health Insurance** **Regulation:** State **Financing:** State **Provision:** Private	**Population size:** regional model **Population description:** frail elderly persons	**Monetary size:** n/a **Existing/Additional funding:** additional **Service included:** broad set of services	No	**Study Design:** Cost-minimisation analysis **Summary of findings:** No difference in life satisfaction.

5. Accountable Care Organisations (ACOs)[11,15,26,35,96]	United States	**Private Health System** **Regulation:** Private **Financing:** Private **Provision:** Private	**Population size:** Various sizes. More than 750 ACOs in the U.S. serve around 20 million people. **Population description:** All the insured individuals in that specific geographical area	**Monetary size:** Capitated budget under a contractual arrangement with an insurer. **Existing/Additional funding: Service included:** a broad set of services	No	**Study Design:** Various evaluations (both qualitative and quantitative). **Summary of findings: mixed findings:** mixed

ACO example: Kaiser Permanente [9]	United States	**Private Health System** **Regulation:** Private **Financing:** Private **Provision:** Private	**Population size:** 8.7 million people in eight regions. **Population description:** All the insured individuals in that specific geographical area	**Monetary size:** Capitation payment. **Existing/Additional funding:** Existing **Service included:** broad set of services.	No	**Study design:** Various evaluations (both quantitative and qualitative) **Summary findings:** one of the top-performing health systems in the U.S.

ACO example: The Veterans Health Administration (V.A.)[9,88]	United States	**Private Health System** **Regulation:** Private **Financing:** Private **Provision:** Private	**Population size:** Regionally based. **Population description:** older people	**Monetary size:** Capitation payment. **Existing/Additional funding:** Existing **Service included:** broad set of services	No	**Study design:** Various evaluations, both quantitative and qualitative **Summary findings:** Reduced hospital bed days by 55 per cent

ACO example: Geisinger Health System [9]	United States	**Private Health System** **Regulation:** Private **Financing:** Private **Provision:** Private	**Population size:** 2.6 million people **Population description:** People with high healthcare needs	**Monetary size:** Bundled payments **Existing/Additional funding:** Existing **Service included:** a broad set of services	No	**Study design:** Various evaluations, both quantitative and qualitative **Summary findings:** increased productivity and patient satisfaction.

6. Gesundes Kinzigtal [58,59,71,90]	Germany	**Social Health Insurance** **Regulation:** Societal actors **Financing:** Societal actor **Provision:** Private	**Population size:** ~35,000 **Population description:** nearly half of the 69,000 residents of the Kinzigtal region	**Monetary size:** shared savings contract based on (virtual) capitated budget **Existing/Additional funding:** additional **Service included:** a broad set of services.	Yes	**Study design:** Propensity Score Matching **Summary of findings:** reduction of 2.5 years in mortality rate.

7. The Arizona Long-Term Care System (ALTCS) [122,123]	United States	**Private Health System** **Regulation:** Private **Financing:** Private **Provision:** Private	**Population size:** all the individuals in a given geographical area. **Population description:** patients at high risk of institutionalisation.	**Monetary size:** capitated budget **Existing/Additional funding:** additional **Service included: a** broad set of services.	No	**Study design:** computer simulation with survey data. **Summary of findings:** decreased nursing home utilisation

8. Better Care Fund (BFC) [8,29,124,125,126,127]	England	**National Health Service** **Regulation:** State **Financing:** State **Provision:** State	**Population size:** 14,362,968 **Population description:** All the patients ehad any planned or emergency hospital admission in the two financial years before the first adoption of the BCF.	**Monetary size: £5.3 billion in** 2015/16, and**£5.8 billion** in 2016/17 for a total of £11.1 billion over the two years. **Existing/Additional funding:** existing funds **Service included a** broad set of services	No	**Study design:** ordinary least squares (OLS) regression and quasi-experimental methods (difference indifference). **Summary of the findings:** no effects on secondary care use for the whole population. Increased use of bed days increased in the short-term by 0.164 (4.9%) per patient per year

9. Norrtalje [42,55,56,57]	Sweden	**National Health Service** **Regulation:** State **Financing:** State **Provision:** State	**Population size:** 65,000. **Population description:** Residents of Norrtalje	**Monetary size:** Capitated budget. Up to 5% of the local authorities’ budget. **Existing/Additional funding: E**xisting funding **Service included:** broad set of services.	Yes	**Study Design:** Pilot, qualitative. **Summary of findings:** Improved coordination but no cost reductions nor improvements in health outcomes.

10. Eksote [128]	Finland	**National Health Service** **Regulation:** State **Financing:** State **Provision:** State	**Population size:** 132,000. **Population description:** Residents of EKSOTE region	**Monetary size: 550 million €** **Existing/Additional funding:** Existing funding **Service included:** all social and healthcare services	Yes	**Study Design:** various studies both qualitative and quantitative. **Summary of findings:** avoid necessary visits and optimized hospital admission


### Health system country context

The vast majority of the international examples of pooled budgets are from private insurance-based health systems. For example, much of the evidence comes from the nearly a thousand examples of Accountable Care Organisations (ACOs) in the U.S. [9,11,12,15,16,17,19,23,26,35,51,85,97,100,118,122,123,129,130,131,132,133]. However, more examples of initiatives to integrate budgets for health and social care are also emerging from Social Insurance/National Health Systems, both in Europe [6,7,8,9,33,35,36,42,48,49,50,55,56,57,58,59,62,63,68,71,90,94,98,99,101,103,117,134] and worldwide [4,9,11,15,16,17,19,23,26,35,51,61,66,74,75,77,79,80,81,83,85,86,87,97,121,129,131,132,133,135,136,137,138,139]. The health system in place in the country where pooled health and social care budgets were implemented seemed to have played a potential role in shaping their form. Initiatives from insurance-based systems appeared to be more oriented towards the provision side (e.g. expanding services delivered towards social care within given budgets), where reforms in National Health Systems involved changes mostly on the funding side (e.g. joining once separately dedicated health and social care budgets to commission services).

### Target population

This review identified two broad categories of pooled budget models. Mostly, pooled budgets targeted a specific sub-population, often people with high healthcare needs [8,9,32,33,35,48,49,50,61,66,68,69,70,74,75,77,79,80,81,83,85,86,87,95,121,122,123,133,135,136,137,139]. In fewer instances, they served an entire population living in a pre-determined geographical area, i.e. ‘place-based’ pooled budgets [42,55,58,59,71,90]. Although often cited as examples of ‘place-based budgets’ [8,9,35,36], ACOs in the U.S. cover defined geography of the integrated provider, but only the insured fragment of that geographical population [11,15,19,35,140]. Norrtalje in Sweden and Eksote in Finland (both with a National Health Service) were the only example of fully ‘place-based’ budgets identified [42,55]]. However, these models covered a relatively small populations, roughly 65,000 in each local authority area in the case of Norrtalje [42,55] and the 132,000 residents of the South Karelia region in the case of Eksote [128]. A hybrid example is represented by Gesundes Kinzigtal, operating what it calls a ‘population-based’ model across half a rural region in the Kinzig Valley in Germany (with a Social Insurance model) [58,59,71,90].

### General structure

Where reported, there was wide variation in the broad aspects we were able to measure, including monetary size [32,79,83,90], whether the budget came from existing [11,15,26,74] or additional funds [32,58,75,90], and the range/comprehensiveness of services covered [32,42,55,56,57]. However, in most cases, the budget appeared to be calculated based on population size [18,68,83], sometimes with additional variable elements determined by payment type, like fee-for-service or bundled payment [9,35].

### Outcomes

We found considerable variation in the range of outcomes reported across the various examples of pooled budgets identified in Table 1. Health outcomes included life satisfaction [61], mortality morbidity and quality of life. In addition, some models reported detailed intermediate clinical outcomes. These included blood pressure, cholesterol and BMI, a measure of secondary care use such as length of in-hospital stay [75], the number of hospitalisations/home hospitalisation [68,83], delayed discharge and nursing home days [68,83]. Among the non-health outcomes, cost per patient [66,86]. In some instances, identifying unmet healthcare needs was an unintended consequence of pooled budgets [15,29]. Other unintended consequences of studies, in PACE, for example, found that some programmes denied access to those with psychiatric or substance abuse problems (‘cream skimming’) [120].

### Historical or existing budgets

The present review identified two broader categories of pooled health and social care budgets. On the one hand, we found examples of initiatives where historical budgets lines were pooled together to co-finance health and social care services, e.g. the UK’s Better Care Fund [8] or Norrtalje in Sweden. On the other hand, we found evidence of models/systems of care that expanded the range of service offered based on pre-existing historical budgets, e.g. ACOs or Gesundes Kinzigtal. For example, ACOs are increasingly shifting their boundaries beyond the traditional definition of healthcare by providing services that align more closely with social services. As above, in the former case, the initiative’s focus was more oriented to the funding side. In the latter, the change mainly happened on the provision side, expanding preventative services offered.

The following sections focus on three illustrative case studies of the closest ‘place-based’ examples identified above, illustrating macro-system differences.

### The Norrtalje Model, Sweden – an example in a National Health Service

In 2006, Norrtalje, a local authority north of Stockholm in Sweden, implemented a single organisation-administered pooled budget for all health and social care for the entire population of circa 65,000. The pooled budget initiative entailed creating a new, single integrated provider organisation that became in charge of delivering the totality of health and social care services. In addition, a joint political board formed by twelve members, six from the local municipalities and six from the county, was to administer the entire organisation, including the right to appoint and dismiss the integrated care provider’s chief executive officer (CEO). What is more, this new organization retains funding responsibilities for the entire population of Norrtaelje and therefore it can be considered an example of a fully place based budget.

Before implementing the reform, the legacy system included the Stockholm county council providing owned and tax-funded primary care and hospital specialist care. Except for two independent family practices, all healthcare personnel were salaried employees of the county council. The Norrtalje local authority, on the other hand, owned and funded local social care, operated a public nursing home, and contracted private home care services. The result was simplified from the previous 40 different contracting agreements between payers and providers to contracting only the integrated organisation through the single pooled budget.

According to a qualitative study [55], fears over the closure of a local hospital were a key motive for implementing the organisational/financing changes in the first place and, as may occur in many relatively small and isolated regions, there is a local spirit that allowed for and drove change – in this case it was known as the Norrtaelje spirit [42]. However, the administrative and pooled budget changes alone were insufficient. Additional barriers such as different working cultures concerns over work boundaries and autonomy, perceptions of extra coordination work still had to be overcome with subsequent projects. The study also reported implementing both organisational/pooled funding changes simultaneously to the service delivery would have faced capacity issues, so they were implemented in phases. Introducing the economic changes also brought much additional administration, having to meet national and county requirements and proving to regulators that the new distribution adhered to the rules for each traditional budget, plus the financing rules of the new integrated joint commissioning board. All considered, it took over five years for any qualitative improvement to patient experience and outcomes to be reported.

### Eksote, Finland – an example in a National Health Service

Established voluntarily in 2008, all the nine municipalities in the South Karelia region in Finland decided to form a joint municipal federation called EKSOTE [128]. Starting from 2010, this new organization, in charge of the whole population budget of €550 million, became responsible for all social and healthcare services in the region. As was the case in Norrtalje, this budget was entirely allocated based on geographical determination, and thus can be considered fully ‘place based’. The services covered by this new established budget include but are not limited to hospital and family services, rehabilitation services, oral and mental healthcare, social services for adults, and special services for the disabled. Eksote is administered by a regional management organization with direct management links with local service providers. The latter pay Eksote a monthly fixed sum. If these financial assets are not sufficient to cover service provisions, Eksote municipal federation cannot ask for additional funds, and it needs to mark a bookkeeping deficit. More specifically, Eksote organization includes Council, an Administrative Board and an Advisory Board of municipality managers. The Council retains the highest decision-making power, including appointing the administrative board and approving financial plans and budgets. Its members are elected from representatives of the municipal councils of the participating municipalities. In addition, an activity-based reimbursement system is in place in Eksote, where providers receive additional resources if they make improvements, such as reducing hospital admission or receiving a penalty otherwise. The underlying principle behind this reimbursement system is to introduce competition across providers to reward high-value care for the region’s residents. To date, some promising examples of integrated services are emerging from Eksote. For instance, the rehabilitative home care service supported independent living by providing multidisciplinary interventions at the older person’s home. As a result, it decreased healthcare and social services utilisation over 24 months compared to usual care, according to the findings of a recent randomized controlled trial [141].

### Gesundes Kinzigtal, Germany – an example in a Social Health Insurance

Gesundes Kinzigtal–“Healthy Kinzig Valley” is an integrated care system located in the Kinzig Valley, south-western Germany [59]. The regional for-profit healthcare management company Gesundes Kinzigtal GmbH administers the whole health care budget for nearly half of the 69,000 residents of the Kinzigtal region. It is a joint venture between health management private for-profit company OptiMedis AG (1/3 of the total shares) and physician Medizinisches Qualitätsnetz—Ärzteinitiative Kinzigtal (literally, Medical Quality Network—Physicians Initiative Kinzigtal), a network of physicians which holds 2/3 of the total shares. A population-based approach, which covers a whole range of health service and beyond for the all the people living in defined geographical area e.g. the Kinzig Valley involves a close collaboration between various actors, including hundreds of providers, general practitioners, specialists and hospitals. Additionally, Gesundes has agreement with local authorities and local private providers, e.g. gyms, sports clubs and self-help groups, to expand the range of services offered beyond the ‘traditional’ health sector by including more preventative and social services. For example, they experimented with a service called ‘Social case management which was supported by social workers and offered support to people with complex social problems and addictive disorders [12].’ Therefore, Gesundes Kinzigtal GmbH is accountable for the entire health care service budget for people of all ages and care needs.

Gesundes Kinzigtal GmbH fosters collaboration with pharmacies, health and sports clubs and local governments. People living in the Kinzigtal valley are insured under one of the two participating sickness funds (which determines population membership within the region). At the heart of the Gesundes Kinzigtal GmbH lies a financial mechanism that aims to improve the margin for the contracting sickness funds. More in detail, the difference in costs sustained by the sickness funds taking part in Gesundes Kinzigtal GmbH and a benchmark calculated by standardising the average costs across all of the (over 100) sickness funds in Germany. This mechanism has been named ‘Virtual Budget’ because Gesundes Kinzigtal GmbH does not directly reimburse providers, but on the contrary, they continue to be reimbursed by their usual sickness fund [58]. However, if a sickness fund spends less for a patient insured with Gesundes Kinzigtal GmbH than it receives from the pool, Gesundes Kinzigtal GmbH will share the difference. A recent evaluation shows, Gesundes Kinzigtal GmbH, achieved a 2.5 years reduction in the mortality rate for those enrolled in the integrated care program [59].

## Discussion

### Summary of the findings

The findings from the scoping review identified very few practical examples of successfully implemented pooled budgets that covered a whole geographical population rather than a population segment. Despite the mapping exercise establishing a considerable number of models of pooled budgets (n = 10) which spanned more than 20 years across seven different countries, the scarcity of information and variability which accompanied their description made it complex to compare them in full. Furthermore, the only three ‘place-based’ examples identified were from localised geographical areas where the macro-environment in which these models have been implemented is likely to have played a significant role in shaping their implementation. For example, the strong sense of community in Norrtalje and the Kinzig Valley (each with a total population of circa 60,000) appears to have acted as a crucial enabler for establishing the pooling of budgets given that both areas have broadly similar geographical characteristics and they both share a non-strictly urban location. Arguably, it would have been different (and probably more difficult) to implement similar radical changes in the way health and social care services are managed and organized in other settings with more open environments, a much larger population, as well as a completely different demographic profile. Furthermore, out of the 22 social and healthcare counties in Finland, only Eksote appeared to have shifted for organizing health and social care services from municipal to a regional level and still for a relatively small population size too. As such, it perhaps represents a unique case rather than the norm. Therefore, the replicability of these models outside of their specific context could be limited.

As mentioned in the introduction of this study, pooled budget funds entail the creation of a single administrative, legal, and organisational system in order to align providers’ incentives and overcome agency problems in the way health and social care is organised and delivered. Therefore, as the path dependency [142] theory also suggests, implementing these changes may be context dependent on a series of contingencies that have occurred in a specific place at a particular point in time. Another set of initial conditions might have produced different outcomes. This has been well documented in the case of Norrtalje, for example, where the integrated system/pooled budget funds met the administrative, legal and organizational system and tradition embedded in the macro-environment but it might also apply to the other two examples of place-based budgets outlined in this review to a certain extent.

### Study findings concerning the wider literature

A direct comparison of the findings from the present review with the previous studies on the subject is complex because none of these studies examined pooled budgets specifically. Instead, they focused more generally on financial integration [2,3]. In addition, even when we retrieved the primary source of information for the models included in our mapping exercise, the wide heterogeneity in how these models were described made comparisons very difficult. As a result, there is a need for more detailed reporting on the specifics of these budgets when they are rolled out to facilitate potential cross-learning. We have attempted to provide an initial framework for this reporting to facilitate this in future reports.

### Strengths and limitations

Adopting a scoping review methodology benefitted this study as the method allowed us to map the literature concerning pooled place-based budgets for health and social care. This literature mapping provided a more precise overview of what pooled place-based budgets are and what conditions might be possible. Furthermore, the flexibility of its approach made it possible to identify models not included in previous reviews, e.g. from the grey literature. While we aimed to be comprehensive in our approach, likely the search strategy did not identify all publications relevant to the subject area. However, the two additional ‘snowballing searches’ in the academic and grey literature strengthened our findings.

Additionally, some models we identified in the search are potentially relevant but did not meet our “sufficient details to determine whether the pooled budget initiatives were practically implemented” inclusion criteria, or were examples where accountability of previously separate budgets remained in place with separate partners (so, not strictly a ‘pooled budget’). Some of these, for instance, were extensive nationwide centralised reforms as in Scotland or New Zealand [143,144]. These centralised reforms deserve further exploration, particularly in terms of potential to incentivise pooled budget innovations, but were out of scope for this review where we focused on those already implemented and described at ground level.

Finally, as we were primarily interested in the descriptions of the budgets themselves, we did not assess the risk of bias of the studies included in the final synthesis, which means equal weight was given to academic and non-academic literature. This indicates that the results of the effectiveness outcomes reported should not be over-interpreted; we were interested in which outcomes were being evaluated at all.

### Implications of the findings for practice and research

An overarching element of a truly ‘place-based’ approach appears to be a close provider overlap with the selected commissioning geography. This might, theoretically, be easier to achieve in a national health system, as seen in Sweden and Finland, where geographical populations can be a planning focus rather than patient self-selection to multiple insurers. One of the other themes of successful implementation of pooled budgets, in the examples of Sweden and even ACOs in the U.S. above, seems to be the simultaneous simplification of the provider landscape into a single/integrated group. Partly, this might quell powerful ‘losers’ as funding flows are changed, usually attempting to shift activity and costs away from large hospitals towards smaller community providers. Without this, it seems alignment of geographical footprints might be, at least, a prerequisite. This might be a problem in some contexts. For example, the NHS outlines primary care networks (PCNs) as a crucial part of ‘place’ in England. Still, a recent analysis of PCN geographies showed “all practices had joined a single PCN in [only] three [of the] commissioning regions” [145]. ‘Ideal’ size of PCNs was set at 30–50 K compared to the circa 250 K commissioning geographies in place at the time, so perhaps not too surprising but with probable implications for contracting and coordination. It becomes more complicated to deal with multiple contracts without this overlap, for example, potentially contracting for proportions of the total population to whom the provider provides its services.

An overarching element that denotes place-based approaches is focusing on proactive care, e.g. prevention, rather than reactive care, e.g. acute and hospital care. However, to the best of our knowledge, at present this is a theme which is relatively under-researched. One of the theoretical foundations behind the pooling of budgets stems from their ability to generate savings while maintaining the same level of quality. As described in the insurance literature, the larger the risk pool, the more predictable and stable the spending is. However, we could not identify any study that evaluated whether this is the case. Furthermore, a key limitation in any literature search is time-lag, which however cannot be resolved in the current paper. In this sense, given the importance of the topic a possible step for further research would be to conduct prospective case studies, with interviews of managers of these case studies to elicit further detail on the way pooled budgets are constructed and implemented. Finally, we did not find any study describing interventions in low/middle-income countries. To conclude, as it often the case when implementing radical organizational reforms there may be unintended consequences associated with them. However, information of the possible unintended consequences associated with the vast organizational changes found in the examples identified by this review were lacking and further research is needed in this direction, as well as to bring together more generalised learning for place based pooled budgets once more studies exist.

## Conclusions

This article reviews practical international examples of pooled health and social care budgets, successfully implemented and evaluated in real-world settings. We investigated further these examples to assess which of them are consistent with the concept of a place-based approach. Two significant conclusions can be drawn from the current research. Firstly, very few examples were fully place-based and covered all the needs of a whole pre-defined geographical area. Secondly, despite their relevance in the current political debate, pooled place-based budgets are still at an early stage of implementation and research. Adequate description is required for future meta-analysis of effectiveness on outcomes.

## Additional File

The additional file for this article can be found as follows:

10.5334/ijic.6507.s1 Appendix. Search strategy.
