# Supplementary material for: Defining Pooled’ Place-Based’ Budgets for Health and Social Care: A Scoping Review
Source: Int J Integr Care. 2022 Sep 13;22(3):16. doi: 10.5334/ijic.6507 (PMC9479665; doi:10.5334/ijic.6507)
Supplement: Appendix. — Search strategy. [file ijic-22-3-6507-s1.pdf]

Appendix

Search strategy

Database search

Ovid MEDLINE(R) 1996 to June Week 2 2020.

Searched on the 26th of June 2020

Text-words (title or abstract)

1. (pool* adj4 budget*).tw. (29)
2. (single payer*).tw. (459)
3. (co-financing).tw.(42)
4. (joint commissioning).tw.(19)
5. (integrat* fund*).tw.(49)
6. (align* budget*).tw. (4)
7. (place-based budget*).tw.(0)
8. (global budget*).tw. (259)
9. (accountable care organization*).tw.(1080)
10. (integrated system* of care).tw.(87)
11. 1 or 2 or 3 or 4 or 5 or 6 or 7 or 8 or 9 or 10 (2031)

MeSH terms

12. Exp Healthcare Financing (898)
13. Exp Budgets/ og [organization & administration]
14. Exp Budgets/ sn [Statistics and Numerical data]
15. Exp "Delivery of Health Care"/ec [Economics] (41215)
16. Exp "Delivery of Health Care"/og [Organization & Administration] (53781)
17. Exp "Delivery of Health Care, Integrated"/ec [Economics] (1751)
18. Exp "Delivery of Health Care, Integrated"/og [Organization & Administration] (6073)
19. Exp social work /ec [economics] (438)
20. Exp social work/og [organization and administration] (2112)
21. Exp state medicine/ec [economics] (39673)
22. Exp Health Facilities/ec [Economics] (29445)
23. Exp state medicine / og [Organization administration] (13348)

24. Exp health care costs /sn [Statistical and numerical data] (12900)

25. Exp Health Care Reform/ec [Economics] (4127)

26. Exp Health Care Reform/og [Organization & Administration] (4976)

27. Exp Economics, Hospital/og [Organization & Administration] (630)

28. Exp Health Policy (84955)

29. Exp Hospitals, public / sn [Statistics and numeric DATA] (3997)

30. Exp Economics, Medical (3814)

31. 12 or 13 or 14 or 15 or 16 or 17 or 18 or 19 or 20 or 21 or 22 or 23 or 24 or 25 or 2 or 27 or 28 or 29 (229541)

32 11 and 31 (1069)

33 limit 32 to (English language and "review articles" and yr="2000 -Current") (92)

Ovid EMABSE 1996 to June Week 2 2020.

Searched on the 26th of June 2020

Subjects Headings

1. exp budget/ or exp financial management/ (357545)
2. health care financing/ (10861)
3. health care delivery/ (143270)
4. social work/ (16848)
5. national health service/ (53042)
6. health care organization/ (99989)
7. social work/ (16848)
8. health care facility/ (56050)
9. health care cost/ (173957)
10. exp health care policy/ or exp health care practice/ (173897)
11. funding/ (45870)
12. health economics/ (21320)
13. evidence based practice/ (64570)
14. information system/ (27319)
15. hospital service/ (10506)
16. hospital cost/ (19547)
17. social care/ (8571)

Text-words same as Medline plus review (no filter in EMBASE)

EconLit from 1996 to June Week 2 2020.

Searched on 26 June 2020

Free text word search (full text, both peer-reviewed and non-peer-reviewed literature, English language)

1. (pool* budget*).tw

2. (integrat* fund*).tw

3. (placed based budget*)

4. (single-payer)

5. (global budgets)

6. (accountable care organisation *)

7. (integrated system of care)

8. (co-financing)

9. 1 or 2 or 3 or 4 or 5 or 6 or 7 or 8 (694)

10. narrow by subjects: health government policy; regulation; public health (140); national government expenditures and health (81); health insurance, public and private (67); state and local government: health; education; welfare; public pensions (39); national budget, budget systems (22); analysis of health care markets (137)

11. final hit count: 242

12. results limited to review articles: 9

Google Scholar from 1996 to June Week 2 2020.

Searched on 26 June 2020

Free text word advanced search using the following terms: pooled budgets, integrated funds, global budget, single-payer, place-based budgets and related terms for each text word. Result limited to review article.

Record Screened: 38 pages

Record included: 31

Grey literature search

The Kings Fund

Record included: 3

SCIE

Record included: 2

Hand-search from included publications: 6

Consultation

The former presentation took place on 8 December 2020 at the Greater Manchester Health and Social Care Partnership executive team meeting. The latter occurred on 20 January 2021 at the Greater Manchester Local Care Organization team meeting.
